# Alpha‐synuclein quantitative seed amplification assay predicts conversion to dementia

**DOI:** 10.1002/alz.71167

**Published:** 2026-01-22

**Authors:** Stefan Bräuer, Verena Sondermann, Iñaki Schniewind, Tom Hähnel, Elisabeth Dinter, Luca Kleineidam, Melina Stark, Matthias Schmid, Sebastian Sodenkamp, Christoph Laske, Eike Spruth, Josef Priller, Daniel Janowitz, Katharina Bürger, Ingo Kilimann, Stefan Teipel, Alexander Storch, Niels Hansen, Jens Wiltfang, Wenzel Glanz, Emrah Düzel, Lukas Preis, Oliver Peters, Julian Hellmann‐Regen, Michael Wagner, Alexander Bernhardt, Johannes Levin, Gabor Petzold, Marie Kronmüller, Anna Gamez, Annika Spottke, Frederic Brosseron, Ayda Rostamzadeh, Frank Jessen, Andreas Hermann, Klaus Fliessbach, Anja Schneider, Björn H. Falkenburger

**Affiliations:** ^1^ Department of Neurology, Faculty of Medicine and University Hospital Carl Gustav Carus Technische Universität Dresden Dresden Germany; ^2^ German Center for Neurodegenerative Diseases (DZNE) Dresden Germany; ^3^ Department for Cognitive Disorders and Old Age Psychiatry University Hospital Bonn Bonn Germany; ^4^ German Center for Neurodegenerative Diseases (DZNE) Bonn Germany; ^5^ Institute for Medical Biometry Informatics and Epidemiology, University Hospital Bonn Bonn Germany; ^6^ Department of Psychiatry and Psychotherapy University of Tübingen Tübingen Germany; ^7^ German Center for Neurodegenerative Diseases (DZNE) Tübingen Germany; ^8^ Section for Dementia Research Hertie Institute for Clinical Brain Research and Department of Psychiatry and Psychotherapy University of Tübingen Tübingen Germany; ^9^ Department of Psychiatry and Psychotherapy Charité Berlin Germany; ^10^ German Center for Neurodegenerative Diseases (DZNE) Berlin Germany; ^11^ University of Edinburgh and UK DRI Edinburgh UK; ^12^ Department of Psychiatry and Psychotherapy School of Medicine and Health Technical University of Munich, and German Center for Mental Health (DZPG) Munich Germany; ^13^ Institute for Stroke and Dementia Research (ISD) University Hospital, LMU Munich Munich Germany; ^14^ German Center for Neurodegenerative Diseases (DZNE) Munich Germany; ^15^ Department of Psychosomatic Medicine Rostock University Medical Center Rostock Germany; ^16^ German Center for Neurodegenerative Diseases (DZNE) Rostock/Greifswald Germany; ^17^ Department of Neurology University Medical Centre Rostock Germany; ^18^ Department of Psychiatry and Psychotherapy, University Medical Center Goettingen University of Goettingen Goettingen Germany; ^19^ German Center for Neurodegenerative Diseases (DZNE) Goettingen Germany; ^20^ Department of Medical Sciences, Neurosciences and Signaling Group, Institute of Biomedicine (iBiMED) University of Aveiro Aveiro Portugal; ^21^ German Center for Neurodegenerative Diseases (DZNE) Magdeburg Germany; ^22^ Institute of Cognitive Neurology and Dementia Research (IKND) Otto‐von‐Guericke University Magdeburg Germany; ^23^ Department of Psychiatry and Neurosciences Charité Universitätsmedizin Berlin Berlin Germany; ^24^ ECRC Experimental and Clinical Research Center Charité Universitätsmedizin Berlin Berlin Germany; ^25^ Department of Neurology, University Hospital of Munich Ludwig‐Maximilians‐Universität (LMU) Munich Munich Germany; ^26^ Munich Cluster for Systems Neurology (SyNergy) Munich Munich Germany; ^27^ Clinic for Vascular Neurology Centre for Neurology University Hospital Bonn Bonn Germany; ^28^ Clinic for Parkinson's, Sleep and Movement Disorders, Centre for Neurology University Hospital Bonn Bonn Germany; ^29^ Department of Psychiatry University of Cologne, Medical Faculty Cologne Germany; ^30^ Excellence Cluster on Cellular Stress Responses in Aging‐Associated Diseases (CECAD) University of Cologne Cologne Germany; ^31^ Department of Neurology Translational Neurodegeneration Section “Albrecht‐Kossel,” University Medical Center Rostock, University of Rostock Rostock Germany; ^32^ Clinic of Old Age Psychiatry and Cognitive Disorders University Hospital Bonn and University of Bonn Bonn Germany

**Keywords:** alpha‐synuclein, Alzheimer´s disease, dementia, Lewy body, real‐time quaking‐induced conversion, seed amplification assay

## Abstract

**INTRODUCTION:**

The alpha‐synuclein seed amplification assay (SAA) has shown excellent performance in the detection of Lewy body pathology in cerebrospinal fluid (CSF). Lewy body pathology is prognostically relevant in patients at risk for dementia. Current assays only provide binary results, so there is a need to quantify the extent of pathology in living patients.

**METHODS:**

In addition to the “standard” SAA, we developed a quantitative SAA (qnSAA) and measured 432 CSF samples (216 baseline–follow‐up pairs).

**RESULTS:**

qnSAA results correlated with cognitive performance. Seventy‐five percent of participants with fast qnSAA kinetics converted to dementia in the observed interval. Overall, participants with fast qnSAA kinetics accounted for 27.3% of dementia converters in the entire cohort.

**DISCUSSION:**

Findings demonstrate promising properties of qnSAA measurements in a cohort of patients at risk for dementia. qnSAA results showed improved prognostic relevance and have potential to measure target engagement of therapies against Lewy body pathology.

## Highlights

- In this study, we investigate the potential of a quantitative seed amplification assay (qnSAA) for alpha‐synuclein pathology in cerebrospinal fluid.
- The qnSAA can resolve clinically relevant differences between patient populations.
- qnSAA results change between baseline and follow‐up, reflecting biomarker progression.
- qnSAA results correlate with cognitive performance.
- qnSAA results allow identification of a patient population at 75% risk of converting to manifest dementia.

## 1 INTRODUCTION

Neurodegenerative diseases are increasingly recognized as syndromes caused by distinct pathogenic factors.^1^ Disease‐modifying treatments require identification of the underlying pathology in living patients. The development of robust biomarkers for amyloid pathology in cerebrospinal fluid (CSF) has paved the way for the first disease‐modifying treatments to be approved for Alzheimer's disease (AD) in the United States and Europe, offering hope to patients with cognitive impairment as their chief complaint.^2^

The pathology of α‐synuclein (aSyn) is generally associated with Parkinson's disease (PD). Indeed, Lewy bodies composed of misfolded aSyn are a neuropathological hallmark of PD and other synucleinopathies, which include dementia with Lewy bodies (DLB).^3^ In addition, aSyn pathology is commonly found in patients with AD, as indicated by its initial description as the non‐amyloid beta (Aβ) component.^4^ The clinical and biological relevance of aSyn pathology in AD is highlighted by its inclusion in the revised criteria for diagnosis and staging of AD.^5^

Lewy pathology can be detected in living patients by aSyn seed amplification assays (SAA), which have shown excellent sensitivity and specificity in CSF from patients with various neurodegenerative disorders, including AD, DLB, and PD.^6^, ^7^, ^8^, ^9^, ^10^ However, there is still an urgent need for a quantitative SAA (qnSAA) that can resolve differences between samples with a distinct load of aSyn seeds. Such quantitative information has the potential to further improve the predictive value of aSyn SAA and assess the effectiveness of therapies targeting aSyn pathology.

In a previous work, we found a correlation of SAA kinetic parameters with cognitive performance in patients with manifest PD or DLB.^11^ In this study, we have further developed our qnSAA concept, which allowed us to investigate two core questions raised by our prior findings. (1) Do patients with dementia suffer from a high burden of aSyn aggregates, or does a high burden of aggregates precede the onset of dementia and potentially cause it? (2) Does the measured load of aggregates change over time, or is it a constant “trait” marker?

## 2 METHODS

### 2.1 Participants

This study includes the 202 participants of the German Center of Neurodegenerative Diseases (DZNE) Longitudinal Cognitive Impairment and Dementia (DELCODE) study and 14 participants of the DESCRIBE (DZNE Clinical Register Study of Neurodegenerative Disorders) study, for which CSF samples were available from baseline (BL) and follow‐up (FU). Both are multicenter, observational studies sponsored by the DZNE.

DELCODE enrolled subjects with subjective cognitive decline (SCD), mild cognitive impairment (MCI), or AD and control subjects without subjective or objective cognitive decline and first‐degree relatives of patients with a documented diagnosis of AD dementia. The general design, population, inclusion/exclusion criteria, and biomaterial sampling procedures of DELCODE have been described elsewhere.^12^ No other additional inclusion/exclusion criteria were applied for DELCODE participants.

**RESEARCH IN CONTEXT**

1. **Systematic review**: We searched in PubMed for other research in the field of alpha‐synuclein “seed amplification assays” (SAA) or “real‐time quaking‐induced conversion” (RT‐QuIC). To place our work in the right context, we cited the relevant literature appropriately.
2. **Interpretation**: We show the clinical and prognostic relevance of quantitative SAA (qnSAA) results in a cohort with participants at risk of dementia and manifest Alzheimer's disease. Kinetic parameters of qnSAA correlate with the cognitive performance and predicted the conversion to manifest dementia with high accuracy (75%).
3. **Future directions**: Our findings need to be confirmed in other cohorts. A greater sample size will allow the analysis of additional existing data, such as magnetic resonance imaging. This will also solidify the potential use in clinical trials—for participant stratification and as a marker for target engagement.

DESCRIBE enrolls subjects with different neurodegenerative conditions. The general design, population, inclusion/exclusion criteria, and biomaterial sampling procedures of DESCRIBE have been described elsewhere.^13^, ^14^, ^15^ We included here participants with a diagnosis of SCD, MCI, or AD.

Overall, we included 216 participants: 66 were healthy controls (HCs) without a diagnosis of a neurodegenerative disease, 91 participants with SCD, 43 with amnestic MCI, and 16 were diagnosed with AD. Available demographic data include age and sex. Aβ42/40 ratio in CSF was obtained using V‐PLEX Aβ Peptide Panel 1 (6E10) Kit (K15200E, Mesoscale Diagnostics LLC). Patients were classified as Aβ pathology positive (A+) based on Aβ42/40 ratio, using a threshold of 0.07.^12^, ^16^ Phosphorylated tau (p‐tau)181 was measured using Innotest Phospho Tau(181P) (81581, Fujirebio Germany GmbH). Patients were classified as tau pathology positive (T+) using a threshold of 71.2 pg/mL.^12^, ^16^

The Global Cognitive Performance Score (neuropsychological testing [NPT] global) was obtained by averaging factor scores obtained for five domains during an extensive neuropsychological test battery (learning and memory, language abilities, executive functions and speed, working memory, visuospatial abilities) as described previously.^12^, ^17^ This score was available for DELCODE participants only.

In addition, 21 patients with clinical diagnosis of DLB were included from the local cohort at Technische Universität Dresden (TUD). DLB was diagnosed according to the revised consensus criteria.^18^ In the TUD cohort, lumbar puncture was performed using standard protocols. CSF samples were centrifuged and frozen at −80°C within 90 minutes after collection. Ethical approval was obtained from Ethik–Kommission der TUD (BO‐EK‐444092021) on October 15, 2021.

### 2.2 Human aSyn monomer production

aSyn protein production and purification was performed as previously described^11^ with minor modifications. For the monomer of the standard SAA, we used an isopropyl β‐d‐1‐thiogalactopyranoside–based auto induction medium to induce aSyn expression in BL21 (DE3) *E. coli* bacteria. For the qnSAA monomer we used a lactose‐based autoinduction medium, resulting in a higher cell density at the end of growth (OD600 ≈ 8.0–10.0 vs. ≈ 14.0–16.0). The subsequent purification protocol was identical for both monomer preparations. In brief, we transformed bacteria with the pET‐28(+) plasmid containing WT‐aSyn with an N‐terminal his‐tag. Cells were harvested after 19.5 hours via centrifugation at 3200 × g for 10 minutes. We resuspended the cell pellet in osmotic shock buffer (400 g/L sucrose, 30 mM TRIS pH 7.2, 2 mM EDTA). After 10 minutes of incubation, the solution was centrifuged at 9000 × g for 30 minutes at 18°C. The pellet was resuspended in water and the suspension centrifuged at 9000 × g for 30 minutes at 4°C. Subsequently, we collected the supernatant and reduced its pH to 3.5 using 1 M HCl. Again, the tubes were centrifuged at 9000 × g for 30 minutes at 4°C. The supernatant was collected and its pH increased to 7.5 using 1 M NaOH. Next, we further purified the protein via immobilized metal ion affinity chromatography using a HisTrap FF‐column (Cytivia) and an NGC chromatography system (BioRad; wash buffer: TRIS 20 mM pH 7.5; elution buffer: imidazole 500 mM in TRIS 20 mM pH 7.5). The fraction containing aSyn was collected and loaded on a HiTrap Q‐HP anion exchange column (Cytivia; wash buffer: TRIS 20 mM pH 7.5; elution buffer: NaCl 1 M in TRIS 20 mM pH 7.5). We pooled the selected fractions and dialyzed the protein against water using a 3.5 kDa MWCO dialysis membrane (Thermo Scientific) at 4°C. In the 17 hour interval, water was changed two times. We measured the protein concentration with a spectrometer (NanoDrop, Thermo Scientific) and stored the protein aliquots at −80°C until further use.

### 2.3 aSyn SAA

The standard SAA was performed as previously described.^11^, ^19^ It has been validated against the SAA at the Istituto delle Scienze Neurologiche di Bologna (ISNB),^11^ which has shown excellent performance in neuropathologically confirmed cohorts.^6^, ^20^, ^21^ Additionally, the standard SAA has been validated against several other groups, one using an assay supplied by Amprion.^19^

In brief, the measurements were performed in a black 96‐well plate (Thermo Fisher Scientific), with six 0.8 mm silica beads (OPS Diagnostics) in each well. The final reaction mix contained 15 µL of CSF added to 85 µL of reaction buffer, which consisted of 40 mM phosphate buffer (Carl Roth) pH 8.0, 0.0015% sodium dodecyl sulfate (SDS; Carl Roth), 10 µM Thioflavin T (Carl Roth), 0.1 mg/mL recombinant aSyn, 170 mM NaCl (Carl Roth). The plate was incubated in a BMG FLUOstar Omega plate reader at 42°C with cycles of 1 minute double orbital shaking (400 rpm) and 1 minute rest. The fluorescence measurements were performed every 45 minutes. Each sample was run in four technical replicates. On every plate, we ran the same two positive and two negative controls. A sample was considered positive if at least two out of the four replicates crossed the fluorescence threshold in 40 hours and negative if no well reached the threshold. A sample with one out of four positive replicates was run again up to three times, a sample that was one out of four for three consecutive times was considered positive. This was the case for one BL sample. The FU sample of the same participant was positive (three of four replicates), consistent with the (borderline) positive result at BL.

For the qnSAA, the buffer conditions were identical to the “standard” SAA, but the aSyn monomer was different. The plate was incubated in a BMG FLUOstar Omega plate reader at 42°C with cycles of 1 minute double orbital shaking (400 rpm) and 1 minute rest, as well. Each sample was run in four technical replicates. On every plate we ran the same two positive and two negative controls. A sample was considered positive if at least one of four replicates reached the fluorescence threshold in 75 hours. The fluorescence threshold was defined as the average intensity of previously measured negative controls during the first 10 hours of recording, plus 40 standard deviations.

To combine information from the four technical replicates of SAA or qnSAA measurements, we defined TT2 (“time to threshold 2”) as the lag phase of the second fastest technical replicate of a sample. For samples with fewer than two out of four positive replicates, the TT2 of the qnSAA was defined as follows: If the standard SAA was negative, then the qnSAA‐TT2 was defined as 90 hours. If the standard SAA was positive and the qnSAA was negative, then the qnSAA‐TT2 was defined as 80 hours. If the qnSAA was positive in one of four replicates, then the qnSAA‐TT2 was defined as (the lag phase of the one positive replicate +90 hours)/2.

To simplify the subsequent analysis of qnSAA data, we classified samples as low seeders or high seeders. The TT2 = 33 hour threshold for this distinction was chosen based on the longest TT2 of qnSAA measurements obtained with CSF from patients with a clinical diagnosis of DLB from the TUD cohort. This is based on the assumption that patients with manifest DLB have a high burden of aSyn seeds.

### 2.4 Modeling of the population decay curve for TT2

We assessed the relationship between mean TT2 (i.e., the mean of BL TT2 and FU TT2) and TT2 slope (i.e., the negative change in TT2 per year) using Pearson correlation and an *F* test to compare a linear fit to a quadratic fit. The TT2 curve was modelled using the following function:

(1) TT2_ij _= *α* * exp(‐*β* * (*t*_ij_+*τ*_i_)) + *c*

where *α* and *β* characterize the cohorts exponential decline, *c* is the asymptotic minimum as *t*→∞, and *τ_i_* is a patient‐specific time shift for patient *i*, aligning individual measurements TT2_ij_ at *t*_ij_ on a common timescale. The subscript *j* indicates the different measurement time points for each patient. For interpretability, the exponential curve was plotted with the highest TT2 value set at *t* = 0. Parameters were estimated via least squares with L2 regularization using a default value *λ* = 0.1, the trust‐region reflective algorithm and a maximum of 20,000 iterations. For robustness, exponential curve fitting was repeated using bootstrapping with 10,000 iterations and calculating median parameter estimates from all iterations. Analyses were performed in Python 3.12 with SciPy 1.13.1 and statsmodels 0.14.2.

### 2.5 Statistical analysis

Data were analyzed using GraphPad Prism (v10.2.2), R Statistical Software (v4.2.1), and Python 3.12 with SciPy 1.13.1. We considered a *P* value ≤ 0.05 statistically significant. To test for normal distribution, the Shapiro–Wilk test was used. Further details on the statistical tests are provided in the respective sections in the main text and figure legends. Because of the limited number of cases, we decided in advance not to test for all assessments that were performed in the cohorts. We focused on a set of parameters for which we expected to find potential differences, based on our previous work and findings from others, as cited in the text. We therefore refrained from correcting for multiple testing.

## 3 RESULTS

### 3.1 Patient characteristics are similar to other cohorts

This study included 216 participants of two DZNE cohorts (DELCODE, DESCRIBE) for which longitudinal CSF samples were available (432 CSF samples). The cohorts included HCs, and people with SCD, MCI, and manifest AD. Participants were enrolled between 2014 and 2020. Demographic data are summarized in Table 1.

**TABLE 1:** Cohort characteristics.

	All	HC	SCD	MCI	Dementia^*^
**Number of participants**	216	66	91	43	16
**Sex (f/m)**	103/113	35/31	39/52	19/24	10/6
**Age at BL in years** ^a^	70.9 (4.8)	71.9 (3.6)	69.7 (5.1)	71.9 (5.4)	70.6 (4.3)
**Education in years** ^a^	14.7 (2.8)	14.7 (2.7)	15.2 (2.9)	14.1 (2.6)	12.9 (2.6)
**Employment in years** ^a^	38.9 (9.5)	39.1 (9.7)	39.6 (8.9)	38.3 (9.6)	35.6 (12.1)
**SAA positive (BL+FU)** ^b^	37 (17.1%)	6 (9.1%)	17 (18.7%)	8 (18.6%)	6 (37.5%)
**Dementia converters** ^b^	22 (10.2%)	0 (0%)	5 (5.5%)	17 (39.5%)	NA
**A−/T−** ^b^	120 (56.6%)	49 (74.2%)	57 (62.6%)	13 (31.7%)	1 (7.1%)
**A+/T−** ^b^	50 (23.6%)	12 (18.2%)	19 (20.9%)	15 (36.6%)	4 (28.6%)
**A+/T+** ^b^	32 (15.1%)	4 (6.1%)	10 (11.0%)	9 (21.9%)	9 (64.3%)
**A−/T+** ^b^	10 (4.7%)	1 (1.5%)	5 (5.5%)	4 (9.8%)	0 (0.0%)
**Total Aβ42** ^a^ **in pg/mL**	8725.9 (2391.7)	9050.3 (2200.3)	8604.0 (2529.3)	8485.3 (2107.8)	8692.7 (3138.7)
**Total Aβ40** ^a^ **in pg/mL**	775.2 (355.3)	862.7 (293.3)	814.0 (381.7)	670.9 (343.6)	416.1 (154.9)
**Total tau** ^a^ **in pg/mL**	416.9 (224.8)	374.9 (150.6)	366.2 (193.1)	480.6 (182.9)	757.5 (425.9)
**Total phosphorylated tau181** ^a^ **in pg/mL**	58.1 (26.9)	52.2 (19.7)	54.6 (24.4)	62.7 (22.4)	95.2 (48.6)

Abbreviations: Aβ, amyloid beta; A+, amyloid beta pathology at baseline; BL, baseline; f, female; FU, follow‐up; HC, healthy control; m, male; MCI, mild cognitive impairment; SAA, seed amplification assay; SCD, subjective cognitive decline; SD, standard deviation; T+, tau pathology at baseline.

^a^Mean (standard deviation),

^b^ *n* (%).

^*^All patients with dementia had a clinical diagnosis of Alzheimer's disease.

For 27 of the 216 participants, CSF samples were positive in the standard aSyn SAA at BL, using the same assay protocol as previously.^11^ Thirty‐six CSF samples were positive at the FU visit; that is, 10 patients switched from a negative SAA at BL to a positive SAA at FU. One participant switched from a positive SAA at BL to a negative SAA at FU. The median time between BL and FU was 2 years. The rate of positive SAA results (12.5% at BL, 16.7% at FU) is in the range of previous reports from similar cohorts.^22^, ^23^

A reduced sense of smell was more common in SAA‐positive participants, as indicated by a lower number of correctly identified sniffing sticks at BL (7.21 vs. 9.50 out of 12, *P* < 0.0001, *t* test). This is consistent with several previous studies.^22^, ^24^ Performance in the Symbol Digit Modalities Test at BL was lower for SAA‐positive participants than for SAA‐negative participants (40.1 vs. 44.7 total correct answers, *P* = 0.036, Mann–Whitney *U* test), consistent with findings by others.^25^ Further, similar to Jonaitis et al., there was no significant difference in the Trail Making Test between SAA‐positive and SAA‐negative participants (TMT part A: 48.9 seconds vs. 50.14 seconds, *P* = 0.72, Mann–Whitney *U* test; TMT part B: 113.4 seconds vs. 115.7 seconds, *P* = 0.82, Mann–Whitney *U* test).^25^

### 3.2 qnSAA kinetic parameters change from BL to FU

The standard SAA has been optimized for the binary distinction between patient samples with and without Lewy pathology. This optimization can make it difficult to resolve differences between individual patients, whereas a broader range of aggregation kinetics can make it easier to resolve differences between samples. To achieve this, we used a modified purification protocol for the aSyn monomer. Indeed, we observed much more variance in lag phases between samples using the qnSAA compared to the standard SAA (Figure S1 in supporting information). At the same time, the results of one specific sample remained reproducible (Table S1 in supporting information). In our hands, the lag phase of the aggregation curve has shown the best correlation with clinical data.^11^ To summarize the individual lag phase values from the two to four positive technical replicates of each measurement, we used the second fastest lag phase (TT2), which better represents the kinetic parameters of a sample than the average lag phase^11^ and showed better reproducibility between laboratories.^19^

**FIGURE 1:**
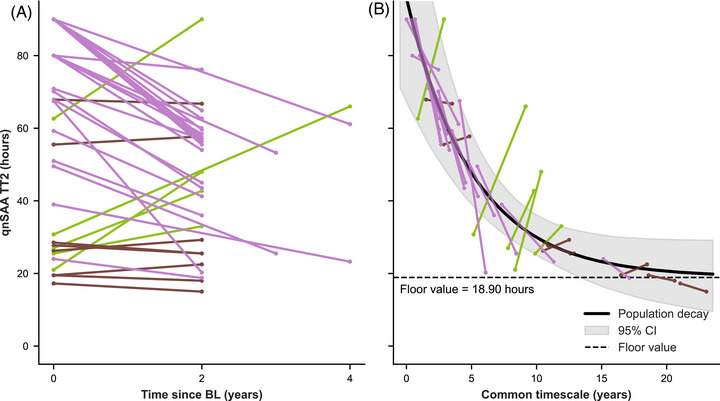
Individual and cohort TT2 decay curves. A, TT2 values for individual patients over time, with each patient's baseline and follow‐up shown by a colored line. Brown indicates minimal change (≤ 3 hours), green indicates an increase (> 3 hours), and lilac indicates a decrease (> 3 hours) of TT2. B, Exponential decay curve (black) modeling TT2 decrease across the cohort. The curve was fitted by modeling a floor effect (dashed line) and aligning individual patient TT2 trajectories (colored lines) to a common timescale. BL, baseline; CI, confidence interval; qnSAA, quantitative seed amplification assay; TT2, time to threshold 2.

We then measured all samples that tested positive in the standard SAA protocol again using the qnSAA. As a first validation, we compared the TT2 between BL and FU of the same patient. Based on the available neuropathological evidence, we expected an increase in the extent of Lewy pathology over time and therefore a higher density of aSyn seeds in CSF.^20^, ^26^, ^27^, ^28^ A higher density of seeds should result in a shorter lag phase.^7^, ^21^, ^29^ Indeed, the TT2 at FU was shorter or equal to the TT2 at BL in 86.5% of participants included in this analysis (Figure 1A). On average, SAA positive participants showed a 14 hour decrease in TT2 between BL and FU. This finding suggests that the qnSAA can report the change in seed concentration that occurs between BL and FU.

We observed a linear correlation between the mean TT2 and the TT2 slope (*r* = 0.50, *P* = 0.0016, Figure S2 in supporting information). There was no evidence for a non‐linear relationship as a quadratic model provided no significant improvement in fit compared to the linear model (*F* = 0.02, *P* = 0.89). Because the exponential function is the only function for which the rate of change is proportional to the absolute value, we can conclude that the TT2 trajectory follows an exponential decay from the linear relationship between absolute TT2 and TT2 slope. We modeled this exponential decay by aligning the TT2 measurements of individual patients in our cohort to a common timescale. This model suggested that TT2 values in our qnSAA decrease toward a minimum of 19 hours, with 90% of the decline occurring within the first 11 years (Figure 1B).

### 3.3 qnSAA seeding identifies patients that subsequently convert to dementia

**FIGURE 2:**
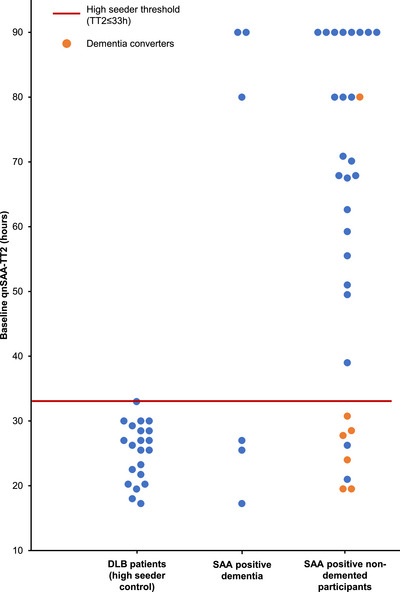
qnSAA‐TT2 results of each SAA‐positive participant in relation to qnSAA‐TT2 of 21 DLB patients from a local cohort. Shown are the qnSAA‐TT2 values for the 21 DLB patients from the local cohort (left column), the qnSAA‐TT2 values of the SAA‐positive patients with manifest dementia at BL from the DZNE cohorts (middle column), and the qnSAA‐TT2 values of SAA positive participants without manifest dementia at BL. Each point represents the qnSAA‐TT2 value of one proband at BL (orange, dementia converters; blue, non‐converters + already manifest dementia). Red line, TT2 threshold (33 hours) to separate high and low seeders. BL, baseline; DLB, dementia with Lewy bodies; DZNE, German Center of Neurodegenerative Diseases; qnSAA, quantitative seed amplification assay; SAA, seed amplification assay; TT2, time to threshold 2.

To facilitate subsequent analysis, we classified SAA‐positive samples into high seeders and low seeders. High seeders were defined as having TT2 values in the qnSAA that are similar to patients with DLB, which show extensive Lewy pathology in neuropathological studies^20^, ^30^ and the most robust SAA positivity in CSF.^6^, ^8^, ^31^ In a local clinical cohort of 21 CSF samples from patients with DLB, the maximum TT2 in the qnSAA was 33 hours (Figure 2). This value was therefore used as threshold to discriminate between high seeders and low seeders.

We then compared the rate of dementia conversion between high seeders and low seeders. In the entire cohort, 22 participants without a dementia diagnosis at BL converted to manifest dementia during the course of the study (11%). The likelihood of converting to a manifest dementia was nine times higher for participants with high seeding in the qnSAA at BL than for all other participants (6/8 = 75.0% vs. 16/192 = 8.3%, *P* = 0.0001, Fisher exact test). It was 11 times higher comparing qnSAA high seeders to low seeders (6/8 = 75% vs. 1/15 = 6.7%, *P* = 0.0017, Fisher exact test). In line with that, dementia converters showed a significantly shorter qnSAA TT2 at BL than SAA‐positive non‐converters (33 hours vs. 59 hours, *P* = 0.018, Mann–Whitney *U* test). Remarkably, qnSAA high seeders represented a disproportionally high share of all dementia converters: 27% (6/22) of dementia converters were qnSAA high seeders, even though only 4% (8/200) of the non‐demented participants at BL (HC, SCD, MCI) were qnSAA high seeders (*P* = 0.0008, Fisher exact test).

Of note, the standard SAA also predicted conversion to manifest dementia. The rate of dementia converters was 3.6‐fold higher in participants with positive aSyn SAA at BL than in participants with negative aSyn SAA at BL (30.4% vs. 8.5%, *P* = 0.0058, Fisher exact test). Moreover, pathological CSF values for Aβ and p‐tau181 predicted conversion to dementia (Aβ: 23.2% vs. 4.6%, *P* = 0.0002; p‐tau 181: 27.2% vs. 7.9%, *P* = 0.0036, Fisher exact test). However, qnSAA high seeding was a more accurate predictor compared to the standard SAA (*P* = 0.0429, Fisher exact test), Aβ pathology (*P* = 0.035, Fisher exact test), and pathological p‐tau181 (*P* = 0.0057, Fisher exact test).

### 3.4 qnSAA kinetic parameters correlate with cognitive performance

**FIGURE 3:**
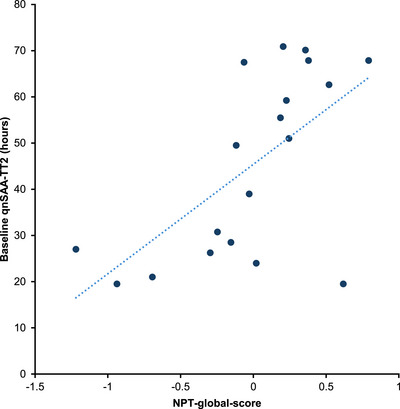
Correlation of qnSAA‐TT2 with cognitive performance. Cognitive performance was quantified by the neuropsychological testing global score at baseline. Each dot represents one participant. NPT, neuropsychological testing; qnSAA, quantitative seed amplification assay; TT2, time to threshold 2.

To further validate the functional impact of our qnSAA measurements, we correlated the cognitive performance as reported by the global score obtained in NPT with qnSAA kinetic features at BL (in participants with qnSAA‐positive CSF). Indeed, we observed a moderate positive correlation between TT2 and the NPT global score (*r* = 0.62, *P* = 0.005, Pearson correlation; Figure 3). Hence, participants with a shorter TT2 (higher expected load of aSyn seeds) showed a poorer performance in the NPT. This is in line with our previous finding in patients with PD and DLB, that patients with a shorter TT2 showed a worse cognitive performance.^11^

### 3.5 Role of aSyn seeding for dementia in the context of amyloid and tau pathology

Next, we separated the prevalence of dementia and the likelihood of converting to dementia during the course of the study based on the presence of Aβ pathology (A+) and tau pathology (T+). aSyn pathology was classified as absent (LB−), low seeder (LB+low) or high seeder (LB+high) using the threshold described above.

**FIGURE 4:**
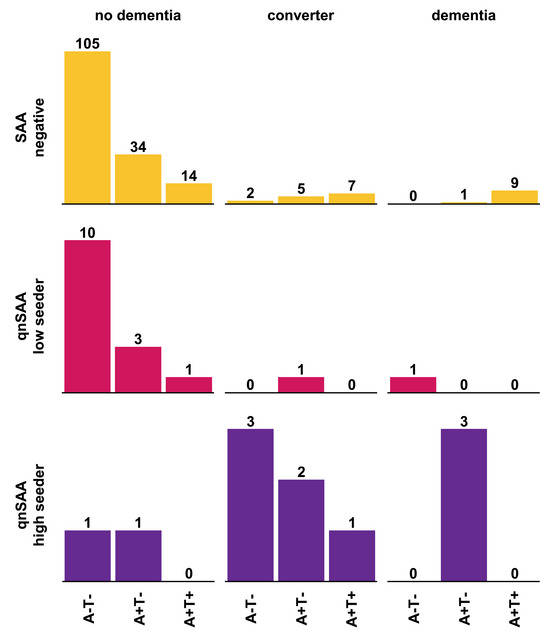
qnSAA seeding in relation to dementia and dementia conversion for patients with different amyloid and tau biomarkers at BL. Number of study participants in each condition. A+, amyloid beta pathology at baseline; BL, baseline; LB+, alpha synuclein pathology as quantified by quantitative seed amplification assay at baseline; qnSAA, quantitative seed amplification assay; SAA, seed amplification assay; T+, tau pathology at baseline.

Overall, 14 participants in this cohort showed manifest dementia and had available CSF BL parameters (Figure 4). Of these, 9 showed the typical A+/T+/LB− pattern and 3 were A+/T−/LB+high. Three of four LB+ cases with dementia were high seeders. In cases with dementia, the rate of A+ was 3 of 4 in LB+ patients and 10 out of 10 in LB− patients, which is not significantly different (*P* = 0.29, Fisher exact test). In contrast, the rate of tau pathology was different between LB+ and LB− cases with dementia: 0 of 4 LB+ cases were T+ whereas 9 of 10 LB− cases were T+ (*P* = 0.005, Fisher exact test). This finding is consistent with the low rate of combined aSyn and tau pathology in previous studies.^22^, ^23^

In participants without Aβ and tau pathology at BL (A−T−), the rate of dementia was low (1/120 participants), and most A−T− participants with high aSyn seeding in qnSAA (3/4 participants) converted to dementia during the observed timespan (Figure 4).

In participants with Aβ pathology but not tau pathology at BL (A+T−), conversion to dementia occurred without aSyn pathology in five of eight cases (Figure 4), consistent with the standard cascade of amyloid pathology known for AD. In addition, three of four A+T− patients with manifest dementia and two of eight A+T− dementia converters were aSyn high seeders. This suggests that even in Aβ‐positive patients, dementia can be associated with Lewy pathology.

In patients with Aβ and tau pathology (A+T+), aSyn seeding was rare, but the sole A+T+ participant with high aSyn seeding converted to manifest dementia (Figure 4).

### 3.6 CSF biomarker profile transition

The availability of CSF samples from two distinct time points allowed us to obtain insight into the sequence of pathological events by observing transitions between biomarker profiles. For 161 of 187 participants, biomarkers were identical between BL and FU (86%). Among the 86 participants with triple negative CSF at BL, 4 cases showed Aβ pathology at FU (5%), 4 cases showed tau pathology (5%), and 6 cases showed aSyn pathology (6%; Figure S3 in supporting information); that is, all three pathologies occurred at comparable rates. Two of 14 participants (12%) with only aSyn pathology at BL acquired Aβ pathology at FU. A small percentage of participants with Aβ pathology at BL acquired aSyn pathology at FU (2 of 49 participants with A+T−LB−; 1 of 23 participants with A+T+LB−). As expected, 6 of 49 participants (12%) with only Aβ pathology at BL acquired tau pathology until FU, and, similarly, 1 of 7 participants (14%) with Aβ and aSyn pathology acquired tau pathology between BL and FU.

## 4 DISCUSSION

In this work, we confirmed that quantitative information obtained from aSyn SAA correlates with cognitive performance. Prospectively, individuals with a short lag phase in the qnSAA (high seeders) were at high risk to develop dementia (75% in the observed time span).

The standard SAA we used was developed by the Caughey and Parchi groups.^6^, ^7^ In patients with Parkinson syndromes, it detects Lewy‐type aSyn pathology with high sensitivity and specificity.^6^ In addition, the assay has been used in the context of cognitive impairment.^8^, ^22^, ^23^ Using this standard assay, SAA‐positive participants showed a 3.6‐fold higher risk of developing dementia than SAA‐negative participants. Consistently, SAA‐positive patients show, on average, a faster cognitive decline.^22^, ^23^

We then optimized the assay to better resolve quantitative differences between individual patients. In this modified assay, the qnSAA, the duration of the lag phase decreased between BL and FU samples in the majority of participants (Figure 1A). This decrease is consistent with the concept that Lewy pathology spreads across the nervous system^28^ and demonstrates that CSF analyses can measure the increased burden of aSyn pathology in living patients. To simplify subsequent analyses, we classified CSF as high and low seeders. However, we assume that the spread of pathology is a continual process (Figure 1B). Accordingly, all 10 participants that were SAA negative at BL and SAA positive at FU showed a long lag phase (upper third) in the qnSAA at FU. This also indicates that there can be floor and ceiling effects in the qnSAA. Indeed, low seeders at BL more commonly showed a decrease in lag phase at FU than high seeders did (81% vs. 9%). The few participants that showed longer lag phase at FU than at BL require further investigation. Factors such as sample processing (e.g., delayed freezing or blood contamination), might contribute to this observation.^32^

Cognitive performance correlated with quantitative information derived from aggregation kinetics (Figure 3). This extends our earlier finding in patients with PD and DLB^11^ to patients with a clinical diagnosis of AD and patients at risk. In PD, similar findings have subsequently been reported by others,^33^, ^34^ and quantitative SAA measurements were found to correlate with the burden of LB pathology as determined by neuropathology.^20^ Collectively, these findings support the notion that differences between samples in the qnSAA are associated with differences in the burden of aSyn pathology in the brain. Several studies demonstrated that the lag phase of the SAA reflects the load of aggregates in the sample.^7^, ^29^ However, the precise relationship between absolute lag phase—as reported by the qnSAA—with the extent and amount of aSyn pathology—as determined by neuropathological examination—still needs to be validated by further studies.

The main difference between the standard SAA and the qnSAA is the preparation of the aSyn monomer. Both preparations are standardized and share the same aSyn sequence. The exact molecular mechanism that mediates the longer run time (75 hours vs. 40 hours) of the assay is subject to ongoing investigations. Some samples were positive in the standard SAA and negative in the subsequent qnSAA (*n* = 5). These samples probably showed a very small load of aggregates, suggesting that the standard assay is more sensitive than the qnSAA. Therefore, the combination of standard SAA and subsequent qnSAA may offers additional value.

Using the qnSAA improved the identification of patients that converted to dementia compared to the standard SAA. Indeed, 6 of 11 (54.5%) aSyn high seeders converted to dementia and 3 already had a manifest (A+) dementia at baseline, so only 2 of 11 (18.2%) patients with qnSAA high seeding remained without dementia during the observed timespan (Figure 4). Therefore, qnSAA high seeders without manifest dementia had a 75% risk of developing dementia in the observed timespan (6 in 8). This risk compares to 6% for aSyn qnSAA low seeders (1 in 15), 30% for all SAA positive participants (7 in 23), 8% for SAA negative participants (15 in 177), 17% for A+T− (8 in 46), and 35% for A+T+ (8 in 23).

Because of their high risk to convert to dementia, aSyn high seeders—as reported by qnSAA—could constitute a very interesting cohort for trials testing novel neuroprotective treatments. In addition, this observation suggests that a relevant subset of patients at risk to develop dementia may benefit from therapies against aSyn. To develop such therapies, the possibility to measure the burden of aSyn pathology through the qnSAA could be used to measure target engagement of therapies directed against aSyn. The striking differences between the biomarker profiles (Figure 4) indicate, however, that patients need to be stratified for Aβ and tau in trials testing therapies against aSyn pathology. Similarly, patients should be stratified for aSyn pathology in trials against Aβ pathology.

The status of aSyn pathology was already included in the revised criteria for diagnosis and staging of AD.^5^ The discrimination between aSyn high seeders and low seeders could further refine this framework. As for other biomarker‐based definition of neurodegenerative diseases,^3^, ^5^ one remaining challenge is the identification of the main or driving pathology compared to co‐pathologies. For instance, Aβ co‐pathology is frequently observed in aSyn‐positive DLB,^8^, ^23^ and aSyn co‐pathology is frequently observed in Aβ‐positive AD.^6^, ^8^, ^35^ The qnSAA could contribute to this distinction. Indeed, five of six A+T− participants with aSyn high seeding were already clinically diagnosed with dementia or converted to dementia. In these tau‐negative patients, aSyn pathology likely contributed significantly to the cognitive impairment.

In this context, the availability of consecutive CSF samples allowed us to investigate sequences of biomarker changes. In patients without any of the three biomarkers at BL, the occurrence of aSyn pathology at FU was about as frequent as the occurrence of Aβ or tau pathology (Figure S2), and this initial pathology could be classified as the main pathology. We observed frequent co‐occurrence of aSyn and Aβ pathology, in the steady‐state analysis (Figure 4), as well as in the transitions (Figure S2). For instance, we observed the occurrence of aSyn pathology at FU in 2 of 49 A+T−LB− patients and the occurrence of Aβ pathology at FU in 2 of 14 A−T−LB+ patients. In contrast, aSyn and tau pathology rarely occurred together without the presence of Aβ pathology—both in the steady‐state analysis (Figure 4) and in the transitions (Figure S2). This observation can be explained by the hypothesis that the triple‐negative state (A−T−LB−) and the Aβ state (A+T−LB−) can be relatively stable, whereas the occurrence of either aSyn pathology or tau pathology are sufficient to trigger neurodegeneration and therefore reduce the time for additional pathologies to occur.

These findings need to be interpreted with caution because of the relatively small numbers in our cohort and they need to be confirmed in additional cohorts. The distinction between main pathology and co‐pathology could be blurred in many cases. This distinction is important, however, for selecting initial therapies in patients at risk for developing dementia. In these trials, it will be interesting to investigate trajectories of aSyn pathology in patients receiving Aβ antibodies and vice versa.

## 5 CONCLUSION

In this study, we demonstrate that the qnSAA can report symptom severity in patients without clinical signs of a neuronal aSyn disease. Furthermore, non‐demented patients with a high burden of aSyn pathology carry a high risk of developing dementia.

## CONFLICT OF INTEREST STATEMENT

All author authors declare no competing interests in regard to this manuscript.

## CONSENT STATEMENT

All participants provided informed consent.

## Supporting information



Supporting Information

Supporting Information
