# Supplementary material for: Alpha‐synuclein quantitative seed amplification assay predicts conversion to dementia
Source: Alzheimers Dement. 2026 Jan 22;22(1):e71167. doi: 10.1002/alz.71167 (PMC12828062; doi:10.1002/alz.71167)
Supplement: Supplementary file 2 — Supporting Information [file ALZ-22-e71167-s002.docx]

Supplementary Figures and Tables for

**Alpha-Synuclein Quantitative Seed Amplification Assay Predicts Conversion to Dementia**

This file includes:

Supplementary Figures S1 to S3

Supplementary Table S1


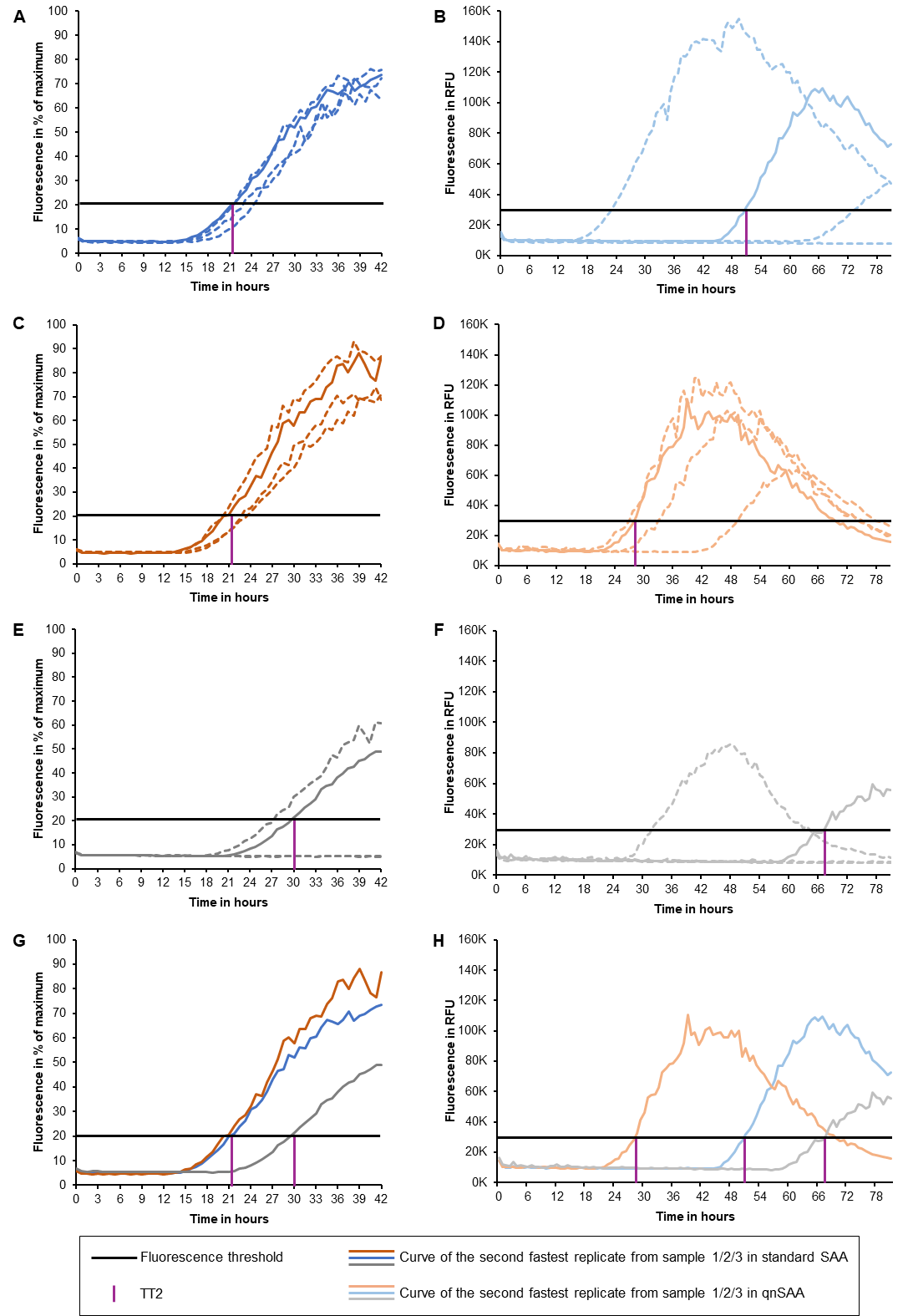


***Supplementary Figure S1. Explanation of TT2 and qnSAA.*** *Depicted are the results of three CSF samples (A+B; C+D; E+F) measured in the standard SAA (A, C, E) and qnSAA (B, D, F). Each sample is measured in 4 replicates. A replicate is considered positive when it reaches a defined fluorescence threshold (black line) in a defined time window (SAA: 40 h; qnSAA: 75 h). The lag phase is defined as the time that a positive replicate needs to cross the fluorescence threshold. The lag phase of the second fastest replicate (solid line) is defined as the TT2 („Time to threshold 2“; purple line). A, C, E show the standard SAA results of the three CSF samples. The curve of the second fastest replicate of each sample in the standard SAA is compared in figure G. Two samples have the identical TT2 in the standard SAA. B,D,F show the qnSAA results of the three CSF samples. The curve of the second fastest replicate of each sample in the qnSAA is compared in figure H. qnSAA TT2 values show much greater differences between samples (SAA: 21.75 h vs. 21.75 h vs. 30 h; qSAA: 28.5 h vs. 51 h vs. 67.5 h).*


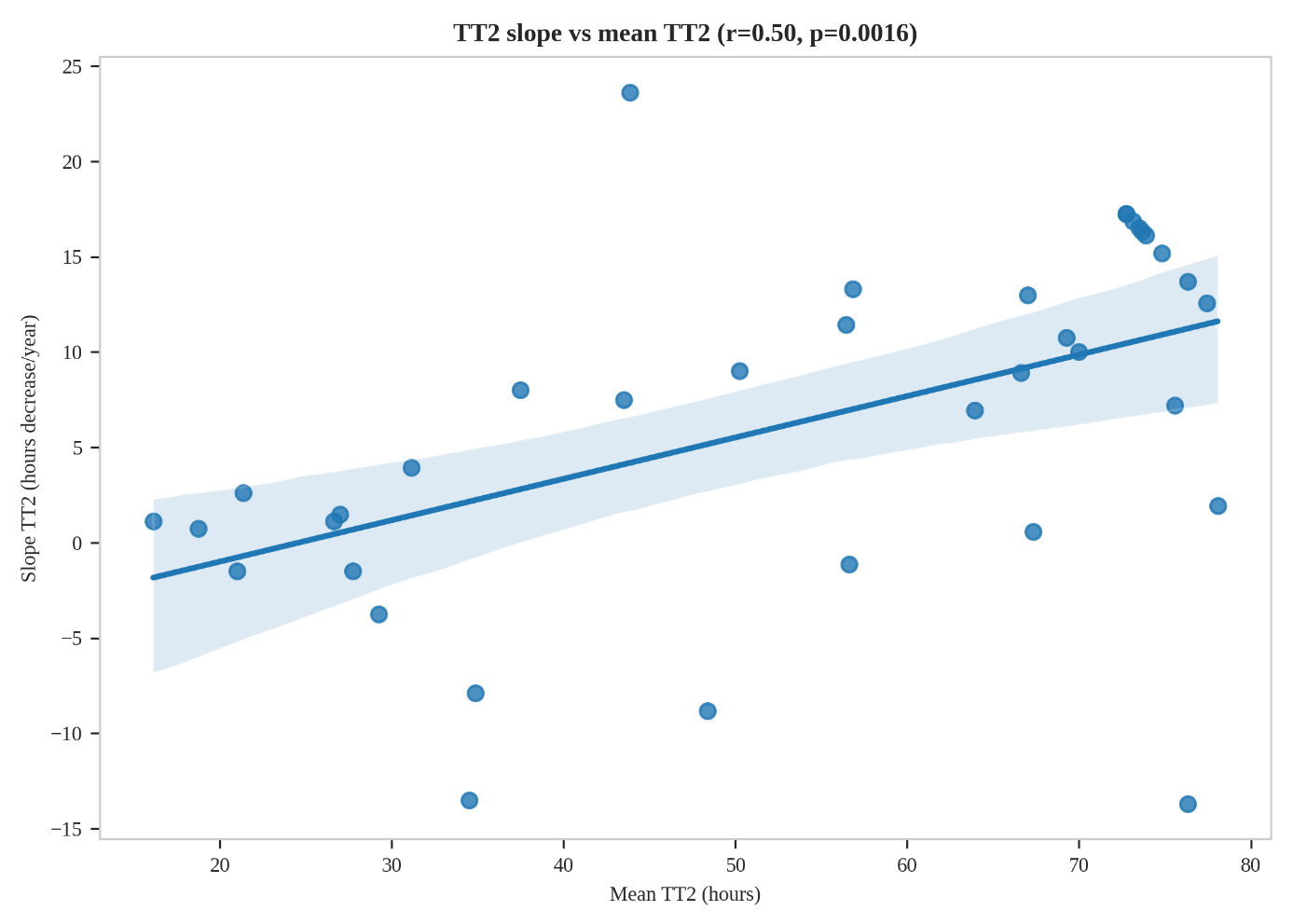


***Supplementary Figure S2. Correlation between TT2 decrease and absolute TT2 values.*** *Shown is the relationship between the TT2 slope (calculated as the change in TT2 between baseline and follow-up, divided by the time interval) and the mean TT2 value (average of baseline and follow-up measurements). The Pearson correlation coefficient, corresponding p-value, and 95 % confidence interval are indicated.*

******

***Supplementary Figure S3. Transitions observed for CSF biomarkers.*** *A+: Aβ pathology, T+: tau pathology, LB+ aSyn pathology as quantified by SAA. Numbers correspond to the percentages of transitions observed between BL and FU, relative to the number of BL samples with that pattern of biomarkers.*

***Supplementary Table S1. Reproducibility of qnSAA TT2 values.***

| Sample | TT2 EXP1 in hours | TT2 EXP2 in hours | TT2 EXP3 in hours | TT2 EXP4 in hours | mean (SD) in hours | Δ(max-min) in hours |
| --- | --- | --- | --- | --- | --- | --- |
| A | 14.25 | 15.0 | 14.25 | 15.75 | 14.8 (0.7) | 1.5 |
| B | 18.0 | 17.25 | 17.25 | 20.25 | 18.2 (1.4) | 3.0 |
| C | 20.25 | 22.5 | 22.5 | 22.5 | 21.9 (1.1) | 2.25 |
| D | 80 | 80 | 71.6 | 80 | 77.9 (4.2) | 8.4 |
| E | 45.75 | 48 | 50.25 | 45 | 47.3 (2.4) | 5.25 |

*Shown are the TT2 qnSAA results of five local samples from clinically diagnosed neuronal aSyn disease (NSD), measured in four experiments. Considering high and low seeder distinction (threshold = 33 h), none of the five samples changes its category with any of its four qnSAA TT2 values.*
